# Sea Clutter Suppression Method of HFSWR Based on RBF Neural Network Model Optimized by Improved GWO Algorithm

**DOI:** 10.1155/2020/8842390

**Published:** 2020-11-12

**Authors:** Shang Shang, Kang-Ning He, Zhao-Bin Wang, Tong Yang, Ming Liu, Xiang Li

**Affiliations:** ^1^School of Electronics and Information, Jiangsu University of Science and Technology, Zhenjiang 212003, China; ^2^College of Underwater Acoustic Engineering, Harbin Engineering University, Harbin 150001, China

## Abstract

The detection performance of high-frequency surface-wave radar (HFSWR) is closely related to the suppression effect of sea clutter. To effectively suppress sea clutter, a sea clutter suppression method based on radial basis function neural network (RBFNN) optimized by improved gray wolf optimization (IGWO) algorithm is proposed. Firstly, according to shortcomings of the standard gray wolf optimization (GWO) algorithm, such as slow convergence speed and easily getting into local optimum, an adaptive division of labor search strategy is proposed, which makes the population have abilities of both large-scale search and local exploration in the entire optimization process. Then, the IGWO algorithm is used to optimize RBFNN, finally, establishing a sea clutter prediction model (IGWO-RBFNN) and realizing the prediction and suppression of sea clutter. Experiments show that the IGWO algorithm has significantly improved convergence speed and optimization accuracy. Compared with the particle swarm algorithm with linear decreasing weight strategy (LDWPSO) and the GWO algorithm, the RBFNN prediction model optimized by the IGWO algorithm has higher prediction accuracy and has a better suppression effect on sea clutter of HFSWR.

## 1. Introduction

High-frequency surface-wave radar (HFSWR) transmits high-frequency (3–30 MHz) electromagnetic waves with vertical polarization antenna, and short waves propagate along the surface of the conductive ocean without being affected by the curvature of the Earth. It can achieve all-weather and over-horizon detection of maritime targets, such as vessels and low-flying aircraft [1–3]. Nowadays, HFSWR is widely used in coastal warning, maritime rescue, marine resource development, and many other fields, and it plays an important role in military and civilian areas [4–6]. Also, a series of innovative research on radar systems made by Ranger, the marine traffic detection EU project, makes the long-distance maritime surveillance radar have a better practical effect [7, 8].

When HFSWR is used to detect marine targets, the resonance effect between electromagnetic waves and ocean waves will produce a large number of interference signals, namely, “sea clutter.” The first-order componen clutter superposing in the echo often submerges some target signals, so the effective suppression of sea clutter becomes the key to the accurate detection of marine targets [9, 10]. The multitarget tracking algorithm based on deep learning has made great achievements in sea target detection [11, 12]. However, from the perspective of signal processing, it is still an urgent problem to eliminate sea clutter from measured signals. At present, the suppression methods of sea clutter mainly include the cyclic iterative cancellation method [13–15], subspace estimation method [16–18], and neural network method [19–21]. The cyclic iterative cancellation method constructs sinusoidal signals by estimating parameters and then subtracts the sinusoidal signals from the echo to realize the sea clutter suppression. The iterative steps of this method are set through experience, which is likely to cause some problems of incomplete suppression of sea clutter and false cancellation of target signals. The subspace estimation method realizes the suppression of sea clutter through its clustering characteristics in the subspace. However, existing suppression methods based on subspace estimation are likely to cause the problem of target spectral peak migration and affect the detection of target signals [22]. The theoretical basis of applying neural network methods is the chaotic characteristic of sea clutter [23, 24]. It is probable to predict and suppress sea clutter accurately if we can learn its inherent dynamics laws. On this basis, the nonlinear prediction equation of sea clutter was proposed by Haykin [25], and scholars want to approximate this complex mapping relationship by establishing neural network models, to realize the prediction and suppression of sea clutter. At present, the deep learning method is booming, and it has made a series of encouraging results in machine vision and engineering applications [26, 27]. In terms of interdisciplinary, deep learning also makes a lot of outstanding contributions with its advantages [28, 29]. Deep learning methods are mainly used in the field of image processing, which needs a lot of training data and a large amount of calculation. This paper aims at the signal field, unlike the image field, which involves a large amount of data, so a shallow neural network is adopted. Because of its simple structure and strong nonlinear mapping ability, the radial basis function neural network (RBFNN) becomes the preference to learn the sea clutter prediction equation. The initial parameters of the network will affect the final prediction accuracy of the model, so it is necessary to optimize them effectively. At present, various metaheuristic algorithms have achieved good results in optimizing the initial parameters of RBFNN [30–32]. The gray wolf optimization (GWO) algorithm was proposed by Mirjalili et al. in 2014 [33], and it has the advantages of fewer adjustment parameters and fast convergence speed [34, 35], compared with other optimization algorithms. However, when facing multimodal functions, it is likely to fall into the local optimal, and its convergence speed and optimization ability are still inadequate [36].

This paper presents a sea clutter suppression method based on an improved GWO (IGWO) algorithm optimizing RBFNN. With greatly improved in convergence speed and the precision of optimization, the IGWO algorithm can significantly optimize RBFNN, which contributes to upgrading the prediction accuracy of sea clutter. Compared with the RBFNN prediction model optimized by the LDWPSO and GWO algorithms, the model proposed in this paper can save calculation time or energy, and it has a better suppression effect on sea clutter.

## 2. GWO Algorithm and Its Improvement

### 2.1. Standard GWO Algorithm

**Figure 1:**
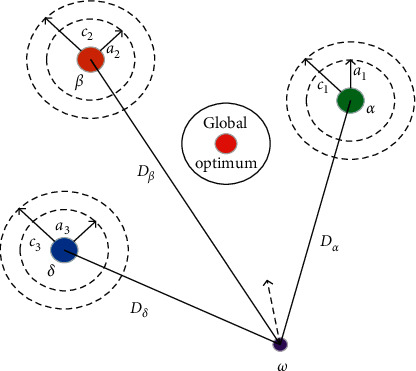
The schematic diagram of gray wolves for optimizing.

The mathematical model of the GWO algorithm is established by imitating the hunting methods of the gray wolf population in nature. During the whole hunting process, all gray wolf individuals are divided into four categories, namely, *α*, *β*, *δ*, and *ω*. Under the leadership of *α*-wolf, the population approaches the prey in an organized way and then surrounds and attacks the prey. In the mathematical model, the position vector of each gray wolf individual is a solution in the solution space. The schematic diagram of the GWO algorithm for optimizing is shown in Figure 1.

Assuming that the population size is N and the dimension of the search space is *D*, the position vector of the *i*th gray wolf is *X*_*i*_ = [*x*_*i*1_, *x*_*i*2_,…, *x*_*iD*_]. The position updating formulas of gray wolves are given by

(1) $$\begin{array}{c} \left\{\begin{array}{c} D_{\alpha }=\left|C_{1}\cdot X_{\alpha }\left(t\right)-X\left(t\right)\right|,\\ D_{\beta }=\left|C_{2}\cdot X_{\beta }\left(t\right)-X\left(t\right)\right|,\\ D_{\delta }=\left|C_{3}\cdot X_{\delta }\left(t\right)-X\left(t\right)\right|, \end{array}\right. \end{array}$$

(2) $$\begin{array}{c} \left\{\begin{array}{c} X_{1}\left(t+1\right)=X_{\alpha }\left(t\right)-A_{1}\cdot D_{\alpha },\\ X_{2}\left(t+1\right)=X_{\beta }\left(t\right)-A_{2}\cdot D_{\beta },\\ X_{3}\left(t+1\right)=X_{\delta }\left(t\right)-A_{3}\cdot D_{\delta }, \end{array}\right. \end{array}$$

(3) $$\begin{array}{c} X\left(t+1\right)=\frac{X_{1}\left(t+1\right)+X_{2}\left(t+1\right)+X_{3}\left(t+1\right)}{3}, \end{array}$$

where **X**(*t*) represents the current position of the *ω*-wolf, **X**_*α*_(*t*), **X**_*β*_(*t*), and **X**_*δ*_(*t*) represent the positions of *α*, *β*, and *δ* wolves, and **A** and **C** are random coefficient vectors:

(4) $$\begin{array}{c} A=a\left(2r_{1}-1\right),\\ C=2r_{2}, \end{array}$$

where **r**_1_ and **r**_2_ are random number vectors between [0, 1] and *a* is the convergence factor. The population can search in any direction around the optimal value because of **A** and **C** [37]. The position vector of the last generation *α*-wolf is the global optimal solution.

In the GWO algorithm, the convergence factors of gray wolves follow the same decreasing strategy. In the early stage, all individuals are used in a large-scale search, which makes the population lack the ability of fine searching and the search space is easily missed; in the later period, all individuals are used in local exploration, which makes the information of the surrounding solution space be ignored, and this is likely to fall into the local optimal. During the entire iteration process, the population cannot search by a division of labor and cannot balance the ability of large-scale search and local exploration, which has a bad effect on the overall performance of the algorithm.

### 2.2. Improved GWO Algorithm

To increase the flexibility of searching, the population is divided into two subpopulations adaptively, implementing different convergence factor strategies, respectively. We set the fitness threshold *ε*, and the population is divided into elite wolves and nonelite wolves by *ε*, and the threshold expression is given by

(5) $$\begin{array}{c} \varepsilon =\mu \cdot \frac{1}{N}\sum_{i=1}^{N}f_{i}, \end{array}$$

where *μ* represents the screening weight, which is used to control the number of elite gray wolves, and *f*_*i*_ represents the current fitness value of the *i*th gray wolf. If the fitness value is less than the threshold, it is classified as an elite gray wolf, otherwise, it is classified as a nonelite gray wolf.

**Figure 2:**
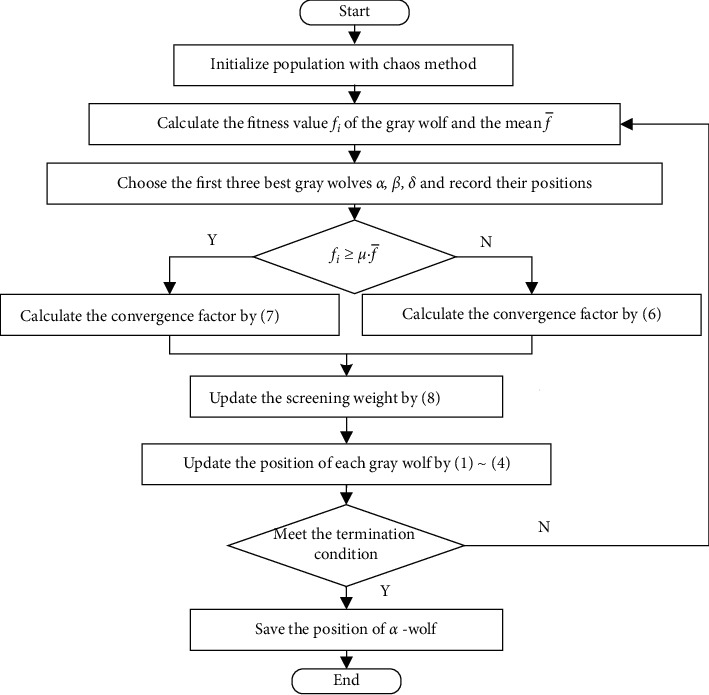
The flowchart of IGWO algorithm.

The elite gray wolves are closer to the global optimum, so the local search should be kept, and the strategy of convergence factor is given by (6); the nonelite gray wolves should keep searching in a wide range because it is far away from the global optimum, and the strategy of convergence factor is given by

(6) $$\begin{array}{c} a_{1}=1-t\cdot \frac{1}{t_{\max}}, \end{array}$$

(7) $$\begin{array}{c} a_{2}=2-t\cdot \frac{1}{t_{\max}}, \end{array}$$

where *t* represents the current iteration step and *t*_max_ represents the maximum iteration steps. To ensure that most individuals can conduct a large-scale search in the early stage and most of them can invest in local exploration in the later stage, we set a dynamic screening weight strategy, which given by

(8) $$\begin{array}{c} \mu =\mu_{\min}+\left(\mu_{\max}-\mu_{\min}\right)\cdot \frac{t}{t_{\max}}, \end{array}$$

where *μ*_max_ is the maximum screening weight, with a value of 0.8, and *μ*_min_ is the minimum screening weight, with a value of 0.2. The IGWO algorithm is given in Figure 2.

The fitness average in each generation is different, and the fitness threshold changes adaptively along with the fitness average. There is always a clear division of labor within the population, which improves the search flexibility.

### 2.3. The Performance Test of IGWO Algorithm

We choose six test functions to test the performance of the IGWO algorithm. To test the generalization ability of the IGWO algorithm, the test functions selected include three categories: *F*1 and *F*2 are unimodal functions; *F*3 and *F*4 are multimodal functions; *F*5 and *F*6 are fixed-dimension multimodal functions. The basic information of test functions is given in Table 1.

**Figure 3:**
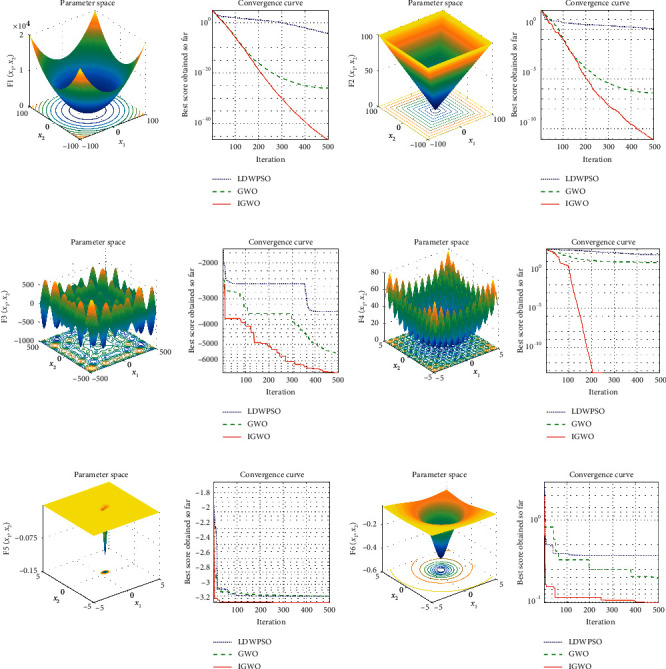
Images of test functions and their convergence curves: (a–f) test functions *F*1–*F*6 and their convergence curves optimized by three optimization algorithms.

Particle swarm optimization is a classic intelligent optimization algorithm with a simple structure and fast search speed. To test the performance of the IGWO algorithm, the particle swarm optimization with linearly decreasing weight (LDWPSO) [38] and the GWO algorithm are used as comparison algorithms. We set the population size to 30 and the maximum iteration steps to 500.  Figure 3 shows the convergence curves of the optimization algorithms in solving test functions.

From the experimental results, the IGWO algorithm has outstanding performance in various test functions. The IGWO algorithm has fast convergence and high accuracy when solving test functions. To eliminate the influence of randomness on the experimental results, 60 experiments are conducted for each algorithm, and the average and standard deviation of the results are taken. The experimental results are given in Table 2.

Analyzing the data in Table 2, among the three algorithms, the results calculated by the IGWO algorithm are closer to the minimum value of test functions, so the IGWO algorithm has higher optimization accuracy. Also, the standard deviation of the calculation results of the IGWO algorithm is significantly smaller than that of the comparison algorithms in all kinds of test functions, which shows that the IGWO algorithm has less volatility and stronger stability.

## 3. Sea Clutter Suppression with IGWO-RBFNN Model

### 3.1. Construction of IGWO-RBFNN Prediction Model

The interior of sea clutter is a deterministic complex dynamical system. If the intrinsic dynamical law of sea clutter can be learned, we can realize the accurate prediction and suppression of sea clutter. To fully demonstrate the internal law of sea clutter, we extend the one-dimensional time series to higher dimensions based on phase space reconstruction theory. On this basis, Haykin proposed the prediction equation of sea clutter [25]:

(9) $$\begin{array}{c} x_{i+m\tau }=f\left(x_{i},x_{i+1},\ldots ,x_{i+m\tau -1}\right), \end{array}$$

where *m* and *τ* are reconstruction parameters of phase space, which represent embedding dimension and time delay, respectively.

**Figure 4:**
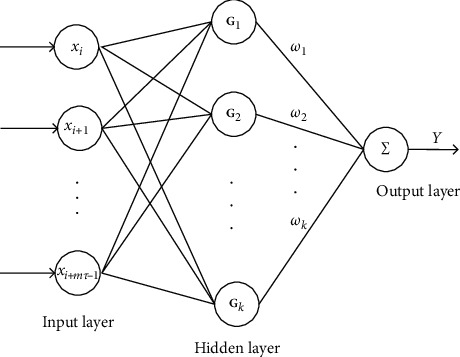
The structure of RBFNN.

We use RBFNN to learn the complex mapping relationship of the prediction equation. RBFNN is a simple three-layer structure, and the input **X** = [*x*_*i*_, *x*_*i*+1_,…, *x*_*i*+*mτ*−1_] and the output is ${\hat{x}}_{i+m\tau }$. Gaussian kernel function maps the input layer to the hidden layer, and the output of the *k*th hidden layer node is shown in

(10) $$\begin{array}{c} G_{k}\left(X\right)=\exp\left(-\frac{{\left\|X-C_{k}\right\|}^{2}}{2\sigma_{k}^{2}}\right), \end{array}$$

where **C**_*k*_ and *σ*_*k*_ are the center and width of the hidden layer node, respectively. The hidden layer and the output layer are connected by the network weight *ω*, and the output of the network is given by

(11) $$\begin{array}{c} Y=\sum_{k=1}^{n}\omega_{k}\cdot G_{k}\left(X\right), \end{array}$$

where *n* represents the number of hidden layers. The network structure of RBFNN is given in Figure 4.

**Figure 5:**
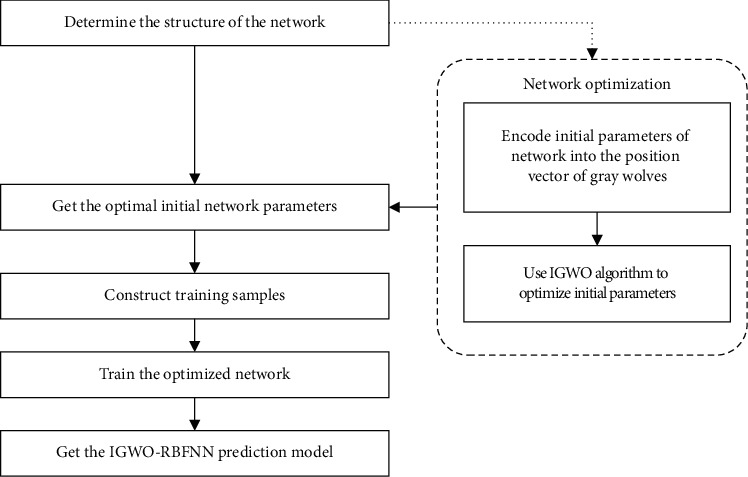
Establishment of the sea clutter prediction model.

The initial parameters of the network include the data center **C**, the data width *σ*, and the network weight *ω*. The selection of the initial parameters will affect the accuracy of the network, so we use the IGWO algorithm to optimize the initial parameters of the network. The fitness function of the IGWO algorithm is given by

(12) $$\begin{array}{c} F=\frac{\sum_{i=1}^{S}{\left(Y_{i}-{\hat{Y}}_{i}\right)}^{2}}{S}, \end{array}$$

where *S* represents the number of samples when calculating the fitness value and *Y*_*i*_ and ${\hat{Y}}_{i}$ represent the expected output and predicted output of the network. The process of establishing the sea clutter prediction model is shown in Figure 5.

  Step 1: determine the topology of the network and encode initial parameters into the position vector of gray wolf individuals
  Step 2: initialize population with the chaos method, use IGWO algorithm to find the optimal solution, and save position vector of the *α*-wolf in the last generation
  Step 3: decode the saved position vector as the optimal initial parameter of the network
  Step 4: normalize sea clutter time series without targets to construct training samples of the neural network
  Step 5: train the network until it reaches the preset precision or the maximum number of training steps and finally obtain the sea clutter prediction model of IGWO-RBFNN

The prediction accuracy of the prediction model is evaluated by *ρ*:

(13) $$\begin{array}{c} \rho =\left[1-\frac{\mathrm{var}\left(\text{err}\right)}{\mathrm{var}\left(Y\right)}\right]\times 100\%. \end{array}$$

In the formula, var(·) represents variance, err represents errors between the output value and observed value, and *Y* represents the observed value of sea clutter.

### 3.2. Suppression of Sea Clutter

**Figure 6:**
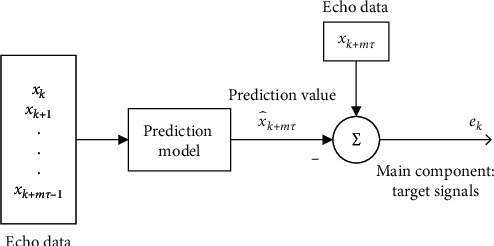
The flowchart of sea clutter suppression.

The sea conditions in the same maritime space are similar, and the internal dynamic system of sea clutter generated by resonance between electromagnetic waves and sea waves at different distances is also similar. We use a prediction model to predict radar echoes of the adjacent unit and then realize the suppression of sea clutter by subtracting predicted value from radar echo data. The suppression process is shown in Figure 6.

The dynamic characteristics of sea clutter in radar echo are different from that of target signals. The prediction model can only predict sea clutter, and the target signal becomes the main component of prediction error. After subtracting the predicted value from radar echo, the target signal is left, and the sea clutter suppression of radar echo containing target signals is realized.

## 4. Experiment and Results Analysis

### 4.1. Analysis of Prediction Effect of Sea Clutter

A batch of HFSWR sea clutter measured data is used for simulation experiments. The operating frequency of radar is 3.7 MHz, and the sampling interval is 0.149 seconds. The radar echo signal is complex, so it is necessary to establish prediction models for the real part and the imaginary part (*I* channel and *Q* channel), respectively. Here, we take the prediction of real data as an example.

**Figure 7:**
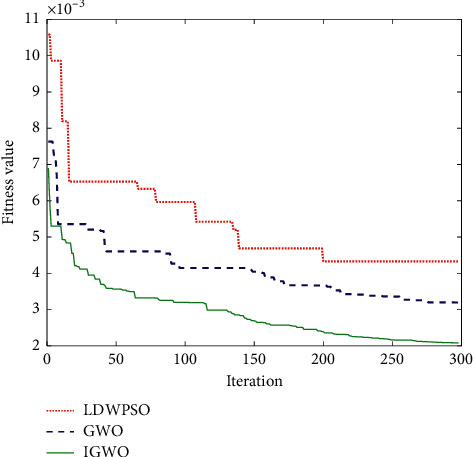
Convergence curves of fitness values.

Reconstruction parameters of phase space are calculated by the C-C algorithm [39], and we get time delay *τ* = 4 and embedding dimension *m* = 3, so the input number of the network is *mτ* = 12. Besides, we set the number of hidden layer nodes to 4. To optimize the initial parameters of the network with different optimization algorithms, we set the population size to 25, the number of iteration steps to 300, and the convergence curve of the fitness value is given in Figure 7.

It can be seen from Figure 7 that the IGWO algorithm has the fastest convergence speed and the highest precision for optimization among three optimization algorithms.

The sea clutter data without target signals in the 48th distance unit is used to construct training data, and 800 groups of training samples are used to train the neural network for 1000 times with an accuracy of 0.0001. The unoptimized network model is the RBFNN model, and the LDWPSO-RBFNN model, the GWO-RBFNN model, and the IGWO-RBFNN model can be obtained through using the LDWPSO algorithm, the GWO algorithm, and the IGWO algorithm to optimize RBFNN. The sea clutter data without target signals in the 54th distance unit is used as test data, and each model is trained independently for 50 times to predict the test data, the experimental results are shown in Table 3. The data compared in the table includes the minimum fitness values (FV) of optimization algorithms, the standard deviation of fitness value (FSTD), the average time used in optimizing network (AVT), and the prediction accuracy (PA) of models.

As shown in Table 3, the minimum fitness value and its standard deviation of the IGWO-RBFNN model are the smallest. The IGWO algorithm has the highest accuracy and the best stability in the optimization of the network. Also, the calculation speed of the IGWO algorithm has been improved. In this experiment, the calculation speed of the IGWO algorithm is 9.94 seconds faster than the LDWPSO algorithm. Because the RBFNN model is not optimized, the prediction accuracy is the lowest. The IGWO-RBFNN has the highest prediction accuracy, which is 8.27% higher than the RBFNN model, reaching 93.49%. The background noise of sea clutter accounts for most of the prediction errors.

### 4.2. Comparison and Analysis of Sea Clutter Suppression Effect

For the data of the imaginary part, prediction models are constructed in the same way. Trained prediction models are used to predict and suppress sea clutter in the 54th distance unit without target signals. The effect of sea clutter suppression is evaluated by the overall decline of echo power and the highest amplitude decline in the Doppler spectrum. The result is shown in Table 4.

**Figure 8:**
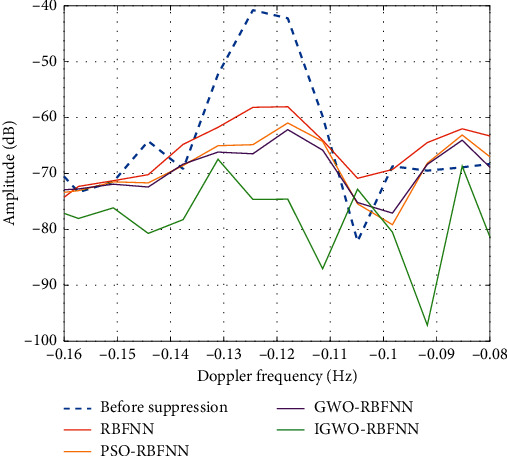
Suppression effect of different models on sea clutter.

In Table 4, the suppression effect of the IGWO-RBFNN model is the best among four models, whether from the perspective of power or the perspective of amplitude. After suppressed by the IGWO-RBFNN model, the maximum amplitude of sea clutter in the Doppler spectrum is reduced by 23 dB. However, after suppressed by the RBFNN model, the maximum amplitude of sea clutter is only reduced by 15 dB, which is 8 dB less than the suppression effect of the IGWO-RBFNN model. Also, the IGWO-RBFNN method reduces the overall power of sea clutter by 83.45%, which is the most prominent among comparison methods. A local magnification of sea clutter suppression effects by different methods is shown in Figure 8.

**Figure 9:**
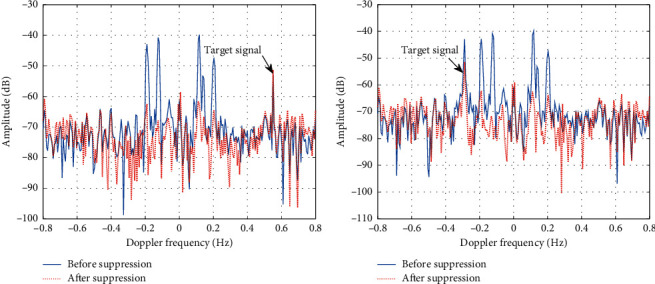
The suppression effect of IGWO-RBFNN model on sea clutter. (a) The distance of Doppler frequency between sea clutter and analog target signal is 0.35 Hz; (b) The distance of Doppler frequency between sea clutter and analog target signal is 0.09 Hz.

It can be seen from Figure 8 that the amplitude of sea clutter is significantly reduced after suppressed by the IGWO-RBFNN model, which is basically equal to the amplitude of background noise. However, after suppressed by other models, the sea clutter still has residual. Next, simulated target signals are added to the radar echo of the 54th distance unit, using the IGWO-RBFNN model to suppress sea clutter in radar echo containing target signals. The distance of Doppler frequency between sea clutter and target signal is 0.35 Hz and 0.09 Hz, respectively. The suppression effect of the IGWO-RBFNN model on sea clutter is shown in Figure 9.

As shown in the calculation results, after using the IGWO-RBFNN model to suppress sea clutter, the signal to clutter plus noise ratio (SCNR) is improved by 5.68 dB and 4.49 dB, respectively. Experimental results show that the IGWO-RBFNN model can effectively suppress sea clutter and retain target signals. After suppressing sea clutter, the target signal can be effectively highlighted.

## 5. Conclusion

According to the sea clutter suppression in target detection of HFSWR, this paper proposes a sea clutter suppression method based on the RBFNN optimized by improved GWO algorithm. Through the establishment of the IGWO-RBFNN prediction model, the sea clutter is predicted and suppressed.

The division of labor search strategy proposed by the IGWO algorithm makes the population have both large-scale search and local exploration capabilities, which improves the convergence speed and reduces the probability of falling into local optimum. In the solution of six test functions, the mean value and standard deviation of the calculation results of the IGWO algorithm are smaller than those of the comparison algorithms, and the accuracy and stability of optimization are improved.

Using the IGWO algorithm to optimize parameters of the network, the calculation time is reduced by 9.94 seconds compared with the LDWPSO algorithm. After the suppression of the IGWO-RBFNN method, the maximum amplitude of sea clutter in the Doppler spectrum is reduced by 23 dB, which is 3 dB more than the optimal result of comparison methods. After suppressing sea clutter of two radar echoes containing target signals by the IGWO-RBFNN method, the SCNR increases by 5.68 dB and 4.49 d, respectively. The above results show that the improved optimization algorithm increases calculation speed. Furthermore, compared with RBFNN, LDWPSO-RBFNN, and GWO-RBFNN models, the IGWO-RBFNN model has a better suppression effect on sea clutter, has better highlight target signals, and provides a good prospect of application in sea clutter suppression.

## Figures and Tables

**Table 1:** Basic information of test functions.

Functions	Dimension	Range	*F*_*min*_
*F*_1_(*x*) = ∑_*i*=1_^*n*^*x*_*i*_^2^	30	[−100, 100]	0
*F*_2_(*x*) = max_*i*_{|*x*_*i*_|, 1 ≤ *i* ≤ *n*}	30	[−100, 100]	0
$F_{3}\left(x\right)=\sum_{i=1}^{n}-x_{i}\sin\left(\sqrt{\left|x_{i}\right|}\right)$	30	[−500, 500]	−2094.91
*F*_4_(*x*) = ∑_*i*=1_^*n*^[*x*_*i*_^2^ − 10cos(2*πx*_*i*_) + 10]	30	[−5.12, 5.12]	0
*F*_5_(*x*) = −∑_*i*=1_^4^*c*_*i*_exp(−∑_*j*=1_^6^*a*_*ij*_(*x*_*j*_ − *p*_*ij*_)^2^)	6	[0, 1]	−3.32
*F*_6_(*x*) = −∑_*i*=1_^5^[(*X* − *a*_*i*_)(*X* − *a*_*i*_)^*T*^ + *c*_*i*_]^−1^	4	[0, 10]	−10.40

**Table 2:** Results of different algorithms for solving test functions.

Functions	PSO		GWO		IGWO	
	Ave	Std	Ave	Std	Ave	Std
*F*1	2.02*E* − 4	3.86*E* − 4	1.34*E* − 27	2.81*E* − 27	7.98*E* − 46	3.97*E* − 45
*F*2	1.15	0.22	6.89*E* − 7	5.91*E* − 7	1.05*E* − 11	1.85*E* − 11
*F*3	−5051.81	1288.74	−5875.91	781.83	−4823.45	666.83
*F*4	54.56	15.99	2.58	3.45	0.80	2.64
*F*5	−3.28	0.06	−3.27	0.07	−3.30	0.05
*F*6	−7.84	3.04	−8.13	2.98	−9.20	2.28

**Table 3:** Results of optimizing models and predicting data.

Models	FV	FSTD	AVT (s)	PA (%)
RBFNN	—	—	—	85.22
LDWPSO-RBFNN	4.30*E* − 3	1.65*E* − 4	72.75	88.92
GWO-RBFNN	3.14*E* − 3	1.53*E* − 4	66.54	89.82
IGWO-RBFNN	2.30*E* − 3	1.06*E* − 4	62.81	93.49

**Table 4:** Suppression effect of different models on sea clutter.

Models	Power decline (%)	Amplitude decline (dB)
RBFNN	81.46	15
LDWPSO-RBFNN	82.21	19
GWO-RBFNN	82.95	20
IGWO-RBFNN	83.45	23

## Data Availability

The data used to support the findings of this study are available from the corresponding author upon request.
